# Life and Death of an Influential Passenger: *Wolbachia* and the Evolution of CI-Modifiers by Their Hosts

**DOI:** 10.1371/journal.pone.0004425

**Published:** 2009-02-11

**Authors:** Arnulf Koehncke, Arndt Telschow, John H. Werren, Peter Hammerstein

**Affiliations:** 1 Institute for Theoretical Biology, Humboldt-Universität zu Berlin, Berlin, Germany; 2 Center for Ecological Research, Kyoto University, Kyoto, Japan; 3 Institute for Evolution and Biodiversity, University of Münster, Münster, Germany; 4 Department of Biology, University of Rochester, Rochester, New York, United States of America; Massachusetts General Hospital, United States of America

## Abstract

**Background:**

*Wolbachia* are intracellular bacteria widely distributed among arthropods and nematodes. In many insect species these bacteria induce a cytoplasmic incompatibility (CI) between sperm of infected males and eggs of uninfected females. From an evolutionary point of view, CI is puzzling: In order to induce this modification-rescue system, *Wolbachia* affect sperm of infected males even though *Wolbachia* are only transmitted maternally. Phylogenetic studies of *Wolbachia* and hosts show that the bacteria rarely cospeciate with their hosts, indicating that infections are lost in host species. However, the mechanisms leading to *Wolbachia* loss are not well understood.

**Results:**

Using a population genetic model, we investigate the spread of host mutants that enhance or repress *Wolbachia* action by affecting either bacterial transmission or the level of CI. We show that host mutants that decrease CI-levels in males (e.g. by reducing *Wolbachia*-density during spermatogenesis) spread, even at cost to mutant males. Increase of these mutants can lead to loss of *Wolbachia* infections, either as a direct consequence of their increase or in a step-wise manner, and we derive analytically a threshold penetrance above which a mutation's spread leads to extinction of *Wolbachia*. Selection on host modifiers is sexually antagonistic in that, conversely, host mutants that enhance *Wolbachia* in females are favoured whereas suppressors are not.

**Conclusions:**

Our results indicate that *Wolbachia* is likely to be lost from host populations on long evolutionary time scales due to reduction of CI levels in males. This can occur either by evolution of single host modifiers with large effects or through accumulation of several modifier alleles with small effects on *Wolbachia* action, even at cost to mutant males and even if infected hosts do not incur fecundity costs. This possibility is consistent with recent findings and may help to explain the apparent short evolutionary persistence times of *Wolbachia* in many host systems.

## Introduction

*Wolbachia* are intracellular bacteria that infect a wide variety of arthropod hosts. Screenings of single individuals per species typically give estimates of around 20% infection [1]–[3], and a recent meta-analysis indicates that the global incidence of *Wolbachia* among insect species can be as high as 65% [4]. As intracellular bacteria inhabiting the cytoplasm, *Wolbachia* are transmitted vertically through female eggs and not through sperm, but can affect phenotypes in both males and females.

As part of their lifestyle as reproductive parasites, arthropod-*Wolbachia* show a wide array of strategies to manipulate hosts into producing higher proportions of infected female offspring. Infected females are favoured because this is the sex that transmits the bacteria to future generations through the egg cytoplasm, whereas *Wolbachia* are not transmitted through sperm. Mechanisms used by *Wolbachia* range from killing of male offspring or induction of parthenogenesis to more subtle manipulations such as generation of sperm-egg incompatibilities, called cytoplasmic incompatibility (CI) [5]–[8]. CI is interpreted to involve a modification-rescue system [6]: *Wolbachia* in infected males modify the sperm, and the same strain of *Wolbachia* must be present in the egg to rescue the modification, or the embryo will typically fail to complete development. Cytologically, CI is manifested as a delay in the nuclear envelope breakdown of the paternal nucleus which results in improper condensation of paternal chromatin in the embryo, typically leading to embryonic death [9]–[11].

The phylogenetic history of *Wolbachia* indicates that long-time phylogenetic concordance between arthropod hosts and *Wolbachia* is uncommon [1], [6], [12]. However, such phylogenetic concordance would be expected due to the vertical transmission of these bacteria typically found within a species. Comparative phylogenetic analyses of *Wolbachia* in nematodes do show concordance with host phylogeny indicative of long term maintenance of the bacteria [13], [14], which contrasts with the pattern observed in arthropod-associated *Wolbachia*. However, *Wolbachia* in nematodes are thought to be mutualistic, whereas those found in arthropods are primarily reproductive parasites (i.e. they manipulate reproduction of hosts to enhance their own persistence). Studies also reveal frequent horizontal transmission of *Wolbachia* between arthropod species on an evolutionary timescale[1], [12], [15]–[17].

Comparing these patterns suggests that *Wolbachia* are frequently acquired by arthropod species, but often do not persist within a species sufficiently long to co-diverge with the host (but see [18] for an exception). There must thus be mechanisms of loss of *Wolbachia*. However, unlike typical infections, resistance to *Wolbachia* infections in females is expected to be selected against, at least for the predominantly found phenotype of cytoplasmic incompatibility. The reason is that infected females are “addicted” to *Wolbachia* – if they lose their parasites, they will be reproductively incompatible with infected males in the population. Therefore, mechanisms by which host species lose their *Wolbachia* despite these antagonistic selection pressures are still unclear, and the male-specific repression of *Wolbachia* that is presented here seems to be one theoretical possibility to solve this paradox.

Early studies on CI-systems elucidated the dynamics of CI-*Wolbachia* and the implications of maternal transmission and infected male incompatibility for the fitness of uninfected females [19], [20]. Previous theoretical studies of the evolution of incompatibility-inducing systems and their dynamics have found selection among parasites and hosts to favor variants that increase the proportion of infected progeny, i.e. by increasing transmission rates [21]. Turelli [21] also addressed possible “resistance” mechanisms in infected or uninfected females: Without assuming mutational costs and given imperfect transmission, he found the spread of modifier alleles reducing susceptibility of infected ova to sperm from infected males [21]. In a similar spirit, previous studies on haplodiploids have examined the spread of costless modifier alleles that completely eliminate or moderately to greatly reduce CI [22], [23], the latter focusing on the effect of different CI types on the spread of such mutations. The dynamics of costly modifier alleles reducing female susceptibility to modified sperm have been examined and suggested as a plausible mechanism to drive nuclear transgenes through insect populations by Sinkins and Godfray [24]. Moreover, a bacterial role in losing *Wolbachia* has been proposed where CI-levels are assumed to slowly degenerate through mutation [25]. CI-inducing *Wolbachia* could also be displaced by sex-ratio distorting mutants, but empirical and theoretical evidence supports this idea only for induction of parthenogenesis and not for male-killing [26]–[28]. However, there has not been a systematic investigation of the effects of male and female specific host modifiers on *Wolbachia* dynamics, particularly treatments that incorporate costs of modifying alleles and consider the consequences to persistence of *Wolbachia* infections.

Here we investigate the co-dynamics of sex-specific host modifiers of CI-*Wolbachia* to determine whether their evolution can explain the lack of persistence of CI-*Wolbachia* in host species. Specifically, we investigate host modifiers that either (a) decrease or increase modification of sperm or (b) decrease or increase transmission of *Wolbachia* through eggs. Previous studies have either focused on – for the case of male “resistance” – the special case of complete elimination of CI through host modification [22] or the special case of CI in haplodiploids (where CI can lead to male production) and the effect of different CI-types on the spread of CI-reducing mutations of moderate to large effect [23].

Our study systematises these approaches but focuses on the long-term fate of *Wolbachia*-infections in the face of host modifier evolution. Moreover, we widen the scope by considering the entire range of putative modifications with relative effects varying from 0% to 100%, and we furthermore introduce survival costs incurred by mutant individuals in order to assess the effect of such costs on the fate of the mutation. We then apply analytical and numerical methods to examine how such regulatory mutants alter *Wolbachia* dynamics and investigate whether such mutations may also cause local extinctions of *Wolbachia*. We find that mutations that decrease *Wolbachia* modification of sperm can often spread and go to fixation in single populations, thereby altering stable infection frequency equilibria or driving *Wolbachia* to extinction, even when elevated survival costs of the mutation are incurred. Moreover, we make explicit use of infection instability to deduce analytically the threshold in effect size above which the spread of a mutation leads to loss of *Wolbachia*. We find that fixation of sub-threshold male-specific repressive mutations (that decrease CI-levels and lower *Wolbachia*-prevalence) eases the conditions for the spread of subsequent mutations of similar kind, so that *Wolbachia* may be lost by way of sequential host adaptation. Mutations that decrease *Wolbachia* transmission or rescue function are disadvantageous to females and never spread for moderate fecundity-costs, while, as expected, enhancing mutations increasing transmission rates are favoured. These modelling results are then discussed within the context of current theoretical, developmental, and evolutionary studies.

## Methods

We investigated the evolution of host modifiers in a single panmictic host population infected with *Wolbachia* that induce cytoplasmic incompatibility. Modification of *Wolbachia* action by the host is controlled by one nuclear locus with two alleles, one being the wild-type, the other the mutation in question. Within the employed haploid model, individuals are characterized by their sex, their genotype at the modifier locus, and their cytoplasmic infection status with *Wolbachia*. Consequently, eight resulting phenotypic classes of hosts are treated in the model. Host-modifier genes are generally assumed to segregate according to standard Mendelian laws. We consider the following classes of host modifiers: (a) enhancement or reduction of *Wolbachia* modification of sperm, (b) enhancement or reduction of *Wolbachia* transmission to eggs, and (c) “mimicry” of egg rescue or sperm modification.

We follow Fine [20] in describing the infection dynamics of *Wolbachia* by two parameters: the transmission rate *t*, and the level of cytoplasmic incompatibility *l*_CI_. The transmission rate is defined as the proportion of an infected female's gametes that contain *Wolbachia*. The level of cytoplasmic incompatibility refers to the non-developing proportion of zygotes that result from fusion of modified spermatozoids with uninfected oocytes. If transmission is complete then the *Wolbachia*-infection will go to fixation, if *t*<1, however, a threshold frequency will exist below which *Wolbachia* cannot invade. Additionally, we assume *Wolbachia* to inflict a fecundity cost on infected females which reduces their offspring number by a factor of 1−*f*. To facilitate comparison with the work of Turelli [21], our transmission rate *t* corresponds to his *1-μ*, our level of CI *l*_CI_ to his *1-H* (or *s_h_*), and our fecundity reduction in infected females *f* to his *1-F* (or *s_f_*).

The mutations under consideration may be grouped according to their sex-specificity (i.e. male- or female-specific) and their effect on *Wolbachia* (i.e. repression or enhancement). Within each class mutations are thought to be sex-specific. Mutations repressing or enhancing *Wolbachia* action in males will alter the modification-function of *Wolbachia* accordingly, while in females such mutations may similarly alter *Wolbachia* transmission-rates (and thereby indirectly the rescue-function of *Wolbachia*) or change the rescue-function directly without affecting transmission. Mechanistically, these effects may either be achieved by affecting gonadal *Wolbachia*-density or by altering *Wolbachia*'s abilities of gamete manipulation.

Moreover, enhancing *Wolbachia* action in the germline could also be accomplished by emulating *Wolbachia*'s modification- or rescue-function autonomously without the presence of *Wolbachia*. A mutation acting in this way would then enable the host to increase modification or rescue without having to interfere directly with *Wolbachia* transmission or manipulation. In this paper, we shall use the term mimicry to describe such an imitation of *Wolbachia* effects. Table 1 outlines the different mutations, groups them according to their sex-specificity and their effect on *Wolbachia*, and specifies mechanistic pathways through which these effects could be achieved. Generally, hypothetical mutations active in males deviate from some previous approaches that focused on mutations decreasing female susceptibility to modified sperm [21], [24]. Moreover, our approach also incorporates conceptually the developmental details of *Wolbachia*-positioning during gamete production and modification [29], [30].

**Table 1:** Hypothetical mutations and their effects.

Effect on Wolbachia	Sex-Specificity	Implementations	Mechanistic Pathways
**Repression**	Male-specific	Lower Modification	Density in Testes
			Efficiency of Modification
	Female-specific	Lower Transmission	Density in Ova
			Efficiency of Transmission
		Lower Rescue	Density in Ova
			Efficiency of Rescue
**Enhancement**	Male-specific	Increase Modification	Density in Testes
			Efficiency of Modification
			Mimicry of Modification
	Female-specific	Increase Transmission	Density in Ova
			Efficiency of Transmission
		Increase Rescue	Density in Ova
			Efficiency of Rescue in Infected Females
			Mimicry of Rescue in Uninfected Females

This table outlines the different mutations examined in our study, groups them according to their effect on *Wolbachia* and their sex-specificity, specifies how these effects could be implemented by hosts, and suggests mechanistic pathways through which these implementations could be achieved.

Phenotypically, we suppose any mutation active in males to result in a different number of spermatozoids being modified by *Wolbachia* and thus to change the effective level of CI to (1−*e*)*l*_CI_. Similarly, mutations active in females are thought to either lead to an altered fraction (1−*d*)*t* of offspring inheriting *Wolbachia* or to a direct change in *Wolbachia*'s rescue function that in turn modifies the effective level of CI to (1−*d*)*l*_CI_. The parameters *d* and *e* represent the mutation's sex-specific penetrance, that is the likelihood of the mutation generating the respective enhancing or repressing phenotype. For reasons of generality, we differentiate penetrance levels *d* and *e* only according to sex-specificity and not to the specifically induced mechanism, and use positive penetrance levels for mutations repressing *Wolbachia* (by reducing CI-levels or transmission rates) and negative penetrance levels for mutations of inverse effects that enhance *Wolbachia* actions through increased transmission rates or CI-levels.

The mutation is further assumed to inflict some costs on affected individuals (in contrast to earlier theoretical studies [21]–[23]), so that the affected sex' chance of survival to adulthood is lessened by a factor of 1−*c* if they carry the mutated allele. Thereby, we hypothesize the mutation's effects on survival to be independent of its penetrance. This cost is motivated by the fact that interfering with bacterial activity might also have influences on the host's own metabolism and vitality – especially if vital host processes co-opted by *Wolbachia* such as microtubule motor protein transport via kinesin-1 [31] need to be modified to, for example, impede bacterial recruitment during gametogenesis.

As shown in the Appendix S1, this verbal description of the system under study may be formalized to give a corresponding mathematical model of eight coupled difference equations. All numbered equations in this text thus refer to the Appendix S1. We were able to deduce analytically thresholds for the penetrance levels of repressive male-specific mutations above which *Wolbachia* is lost, but did not treat the general model analytically. Hence, we employed computer simulations to analyse the dynamics of the model for a wide range of parameters. For every simulation, we used the analytically derived equilibria of the mutant-free system as a starting point and then introduced the respective mutation in all cytogenotypes at a combined starting frequency of 0.1%. Simulations were performed using C++ and the DevC++-compiler (Bloodshed Software), and simulations were continued until equilibria were reached where frequency changes per generation were less than 10^−7^. Analytical calculations were performed by hand and using Mathematica (Wolfram Research Inc.).

## Results

For computer simulations, we considered large parts of the multi-dimensional parameter space. Transmission rates are usually high in nature [32]. Here, we let transmission rate *t* vary between 0.9 and 1. Guided by empirical studies showing CI levels to be variable [33], we let the level of cytoplasmic incompatibility *l*_CI_ take on values across the whole parameter range (i.e. between 0 and 1). We used an upper limit of 50% for the fitness cost of modifier alleles, supposing higher values as very unlikely for an effect of this kind. For the respective mutations' levels of penetrance (*e* in males and *d* in females) we chose values from the entire meaningful range.

Fixed point analysis of the system without mutants reveals a threshold CI-level relative to the transmission rate below which a *Wolbachia* infection cannot persist and disappears from the population (see eqs. 10–12 in Appendix S1). We replicate the results of Fine [20] in showing this threshold – in the absence of fecundity costs to infected females – to be 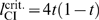 (for only then the relevant fixed points are non-negative and non-imaginary). This threshold value is pivotal in determining whether the spread of a modifier allele causes the extinction of *Wolbachia* and was confirmed by simulation.

### Male-specific mutations

Here, we consider mutants active in male hosts that change the level of CI (i.e. by regulating *Wolbachia*-density during spermatogenesis or reducing *Wolbachia*'s ability to modify sperm) and consider whether these can spread in an infected population. This concept draws on an idea already suggested by Turelli [21]. The concept of male-specific modifiers was put forward verbally by Charlat et al. [14], and the idea of host-mediated changes in CI-levels has been developed formally for the special case of complete elimination of CI by Vala et al. [22] and for different CI-types in haplodiploids by Vavre et al. [23]. Our study systematically enlarges on these concepts and shifts the focus to the evolutionary fate of *Wolbachia*-infections. We allow for the whole range of host modification with effects from 0% to 100% and further introduce a survival cost incurred by those males carrying the mutation in question. Further, we consider the sequential evolution of male and female modifiers on stability of the *Wolbachia* infection.

#### Repressive mutations in Males

**Figure 1:**
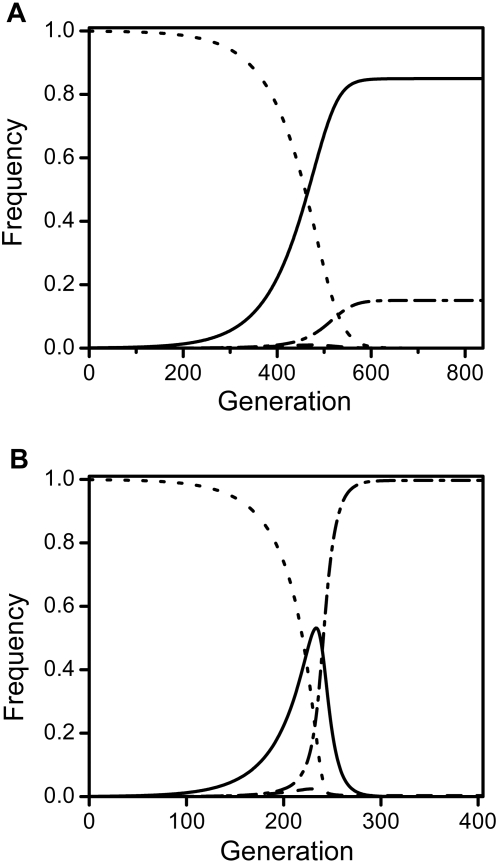
Spread of male-specific cost-free repressive mutations. This figure illustrates the two scenarios of the spread of a male-specific repressive mutation with *Wolbachia* either persisting (graph 1a) or going extinct (graph 1b). The graphs show the frequency of the four different classes: Dashed lines represent uninfected wild-types and dash-dot lines uninfected mutants while infected wild-types are depicted by dotted lines and infected mutants by solid lines. Penetrance levels were varied between *e* = 0.5 in subfigure 1a and *e* = 1 in subfigure 1b. Other parameters were *t* = 0.9, *l*_CI_ = 1, *f* = 0, and *c* = 0.

In the absence of survival costs, male-specific repressive mutations always spread as long as transmission is imperfect (*t*<1; as shown analytically in eqs. 31&32 of Appendix S1). An important finding is that spread of an incompletely repressing mutation can lead directly to the elimination of the *Wolbachia*-infection, or to a lower equilibrium frequency, depending primarily on the penetrance *e*. These contrasting outcomes are illustrated in figure 1, where subfigures 1a and 1b show the mutant's invasion dynamics for different penetrance levels *e*. Specifically, subfigure 1a presents the case of *Wolbachia*-persistence at reduced prevalence levels whereas subfigure 1b depicts the scenario of *Wolbachia*-extinction. Generally, as the mutation spreads among infected individuals, the frequency of uninfected individuals increases as well, and the selective advantage of mutant males correlates positively with the amount of uninfected females present as well as with the value of *e*.

As the mutation rises in frequency, *Wolbachia* experience a new “effective” CI level that is equivalent to (1−*e*)*l*_CI_ at fixation of the mutation. Consequently, *Wolbachia* disappear if the mutation reduces the population's “effective” CI level to values below the threshold 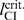 (as derived above) where the *Wolbachia*-infection cannot stably persist. Thus, a threshold for *e* exists relative to *l*_CI_ and *t* above which the successful spread of a regulatory mutation drives *Wolbachia* to extinction. This threshold's exact value can be calculated by setting (1−*e*)*l*_CI_ equal to 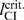 to give 
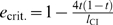
. These analytical results were confirmed by simulation and are summarized in table 2.

**Table 2:** Analytical results.

Survival Costs	Success of Male Mutant	Consequences for *Wolbachia*
*c* = 0	Mutant spreads if and only if *e*>0 given *t*<1.	*Wolbachia* extinction if and only if 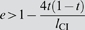 .
*c*>0	Mutant spreads if and only if 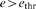 (see Appendix S1).	*Wolbachia* extinction if and only if 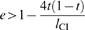 .

This table summarizes the analytically calculated thresholds for the spread of a male-specific repressive mutant as well as the consequences of such a mutation's spread for the persistence of *Wolbachia*.

When *e* is below this threshold, however, the repressive mutation's spread does not lower the population's “effective” CI level sufficiently for *Wolbachia* to disappear from the population. Thus, a new equilibrium of reduced *Wolbachia*-prevalence is attained (see figure 1a). The actual equilibrium values depend on the value of *e*: As *e* grows, a lesser proportion of mutants remains infected in the subsequent equilibrium. The exact new prevalence levels can be calculated analytically by replacing *l*_CI_ in the steady states of equations 10 and 11 in Appendix S1 with 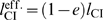 (see equations 33 and 34 of Appendix S1).

**Figure 2:**
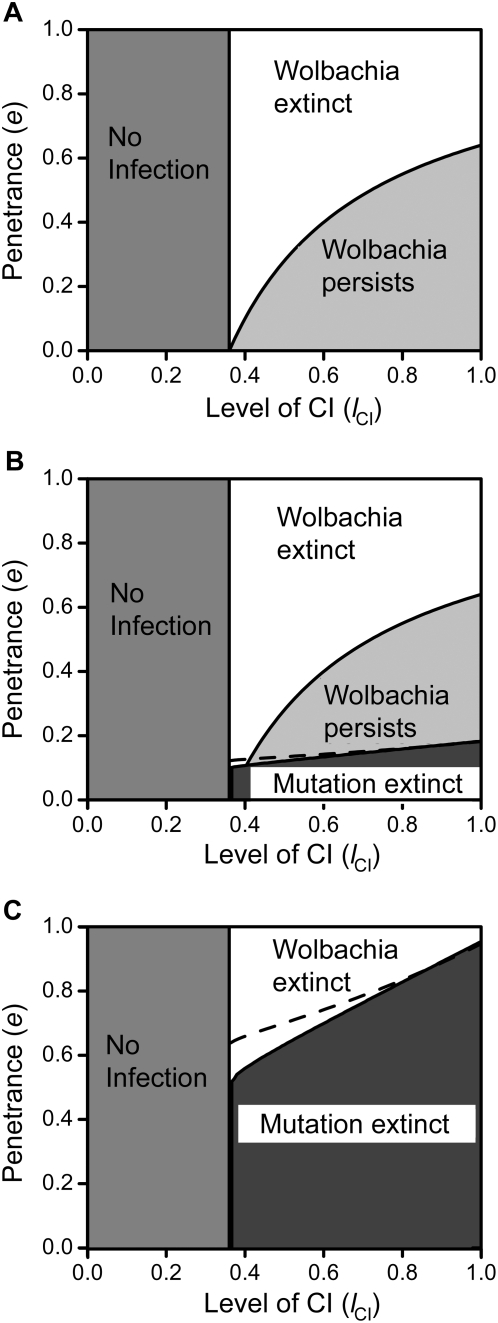
Parameter regions of spread of costly male-specific repressive mutations and loss of *Wolbachia*. Shown are the parameter regions of a mutation's penetrance levels *e* and the level of CI *l*_CI_ where a male-specific repressive mutation can spread in the population, and how this affects the persistence of *Wolbachia*. In 2a the survival cost was set to *c* = 0 and the mutation could always spread. In 2b and 2c the survival cost was set to *c* = 0.01 and *c* = 0.05, respectively. Dashed lines mark the critical penetrance *e*_crit._ as approximated analytically in eq.30 of Appendix S1. Other parameters were *t* = 0.9, *d* = 0, and *f* = 0.

When we introduce a survival cost *c* that is inflicted on mutant males, the repressive mutation's penetrance *e* needs to be above a certain threshold *e*_thr_ for the mutation to spread. This threshold depends on the original CI-level *l*_CI_, the transmission rate *t*, and the survival cost *c*. An analytical approximation of *e*_thr_ can be derived by taking into account the reduced cost of CI incurred by mutants and then projecting the population dynamics one generation into the future (see figure 2 in the main text and equation 30 of Appendix S1). However, infected mutant females, despite being phenotypically indistinguishable from the wildtype, also enjoy a reproductive advantage through indirect fitness effects due to the production of infected mutant sons. When projecting the population dynamics only one generation into the future, mutant benefits therefore are underestimated (and threshold levels *e*_thr_ overestimated as a consequence, see figure 2). This estimation error can, for example, be attenuated by projecting the population dynamics one more generation into the future (see figure 1 of Appendix S1).

As in the costless case, the mutation's successful spread leads to two possible scenarios: Either it causes the extinction of *Wolbachia* or the mutation spreads but *Wolbachia* persist at reduced prevalence levels. Again, the consequences of the mutation's spread depend on the level of *e*: The analytical threshold value *e*_crit._ derived above is confirmed by the numerical results and does not vary with survival cost *c*:While costs do influence whether a mutation invades or not (see below), they do not alter the qualitative dynamics where invading mutations always go to fixation, so that *e*_crit._ remains a function of only *l*_CI_ and *t*.

**Figure 3:**
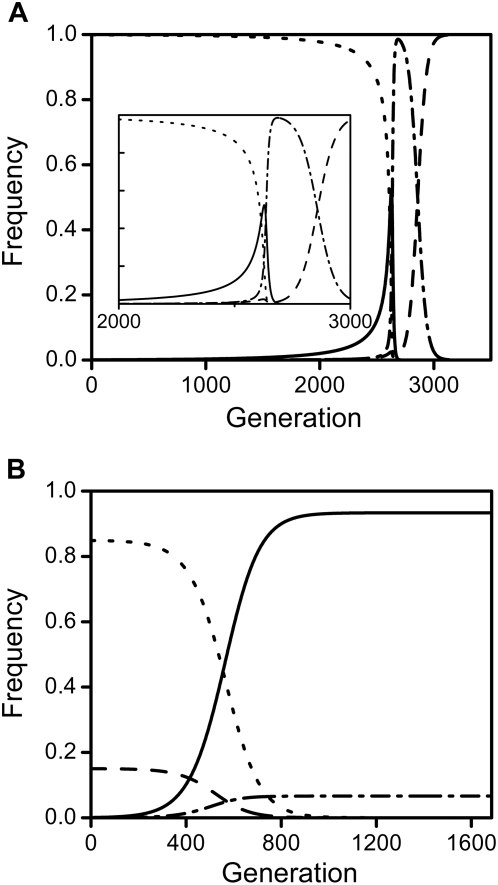
Spread of costly male-specific repressive and cost-free female-specific enhancing mutations. Shown are typical dynamics for the spread of a male-specific repressive mutation with associated survival costs and *Wolbachia* going extinct (3a, insert enlarged for temporal clarity), or of a female-specific enhancing mutation increasing transmission rates without survival costs (3b). Dashed lines represent uninfected wild-types and dash-dot lines uninfected mutants, while infected wild-types are depicted by dotted lines and infected mutants by solid lines. Parameters were *t* = 0.9, *e* = 1, *l*_CI_ = 1, *f* = 0, *c* = 0.05, and *d* = 0 in 3a and *t* = 0.9, *e* = 0, *l*_CI_ = 0.5, *f* = 0, *c* = 0, and *d* = 0.05 in 3b.

Typical dynamics of the spread of a costly mutation with *Wolbachia* being lost from the population are depicted in figure 3a. As in the case without costs, the repressive mutation increases in frequency until average CI levels are lowered to 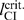 (see above). Thus, the infection becomes unstable, *Wolbachia* suddenly disappears, and only uninfected hosts remain. However, with all females being uninfected, the survival cost incurred by mutant males is no longer balanced by their increased reproductive success. Thus, in contrast to the costless case presented in figure 1b, the mutant allele disappears from the population (as demonstrated for a female-specific costly “nuclear rescue construct” by Sinkins and Godfray [24]).

**Figure 4:**
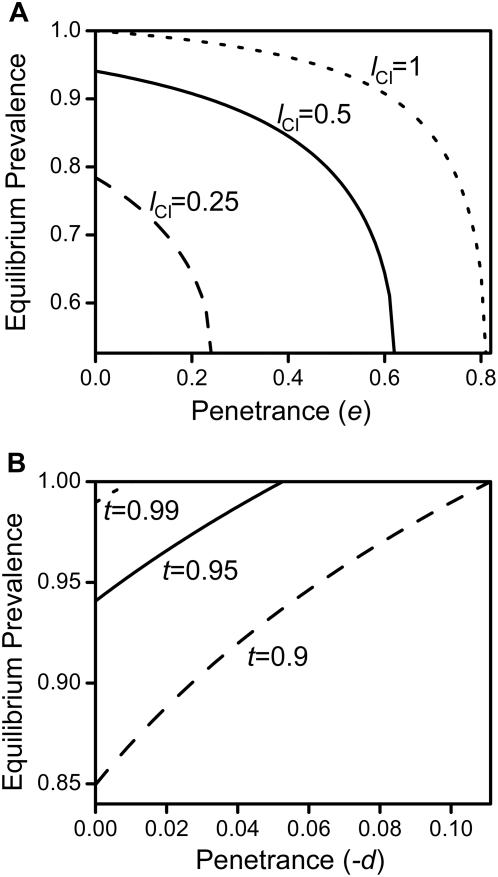
Altered *Wolbachia*-prevalence after spread of repressive and enhancing mutations. Shown in 4a is the reduced prevalence of *Wolbachia* after successful spread of a male-specific repressive mutation. Prevalence is shown as a function of the mutation's penetrance *e* for different values of *l*_CI_ (as indicated in the graph) and with *t* = 0.95. Figure 4b shows the elevated prevalence of *Wolbachia* after fixation of a female-specific enhancing mutation as a function of the mutation's penetrance −*d* for different values of *t* (as indicated) with *l*_CI_ = 0.5. All plots are based on the analytical results of eqs. 33&34 and eqs. 35&36 respectively, all in Appendix S1. In 4a, *e* was varied between *e* = 0 and *e* = *e*_crit._ for each case. At higher values of *e*, the spread of the mutation reduces *Wolbachia*'s prevalence to zero. In 4b, −*d* was varied between −*d* = 0 and 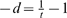, as larger values of −*d* all lead to *t* ^eff.^ = 1. Other parameters were *c* = 0 and *f* = 0.

If the mutation does not drive *Wolbachia* to extinction but only reduces the bacteria's prevalence, the resulting dynamics are qualitatively similar to the costless case presented in figures 1a and 1b. As a consequence, the costly mutation persists at a new equilibrium of infected and uninfected mutants. The exact new prevalence levels can be calculated analytically as in the costless case (this is illustrated in figure 4a). However, sex ratios are skewed when *c*>0, as costs are only incurred by males. Thus, the analytical predictions of equations 33 and 34 in Appendix S1 only hold for the overall prevalence of *Wolbachia* but not for the individual equilibria of males and females.

**Figure 5:**
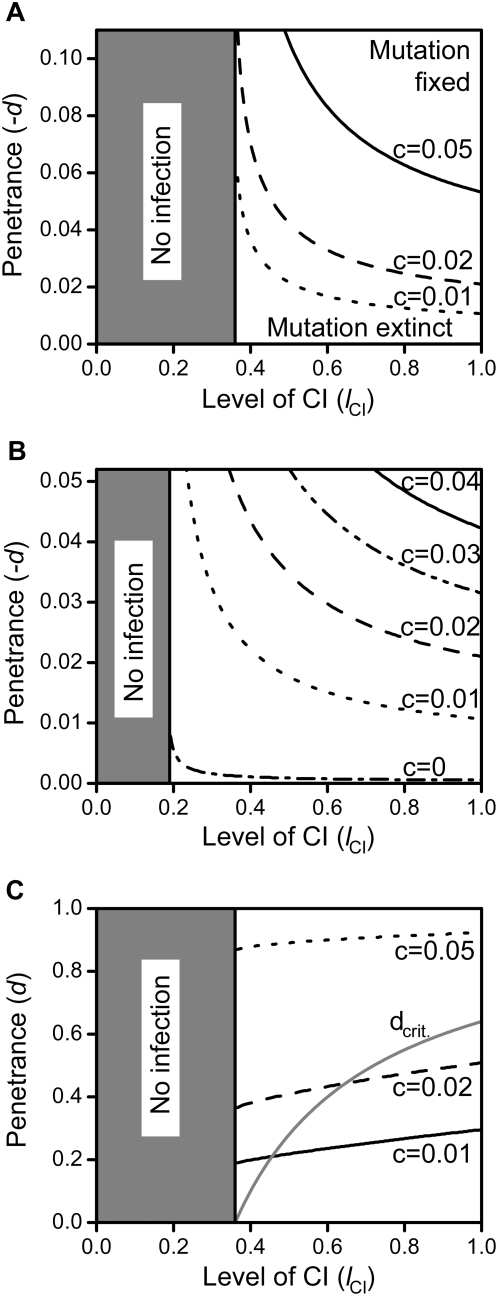
Parameter regions of spread of female-specific enhancing mutations. Shown are the parameter regions of the mutation's penetrance levels *d* and the level of CI *l*_CI_ where a female-specific enhancing mutation that either increases transmission rates (5a and b) or increases the rescue function autonomously (5c) can spread in the population. Transmission rates were varied between *t* = 0.9 (5a and c) and *t* = 0.95 (5b). The survival cost *c* was varied as indicated, and the mutation could invade (and spread to fixation) above the depicted threshold lines. In 5a and c, costless mutations could always invade. Penetrance levels in 5a and b (where they take on negative values in order to increase transmission rates) were varied between −*d* = 0 and 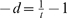 (where *t* ^eff.^ becomes 1) and between 0 and 1 in subfigure c. The grey line in 5c depicts the critical penetrance level *d*_crit._ above which *Wolbachia* is driven to extinction by the mutation's spread. Other parameters were *e* = 0 and *f* = 0.

In general, the mutant allele's invasion success is influenced by the CI-level 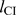 and the mutation's penetrance *e*, as well as the survival cost *c* and the transmission rate *t*. Figure 2 shows the dependence of invasion success on *l*_CI_ and *e* for different values of *c*. These results show that successful invasions require higher levels of penetrance as survival costs increase, since the mutational benefits need to outweigh the associated rising fitness costs. Moreover, if transmission rates rise and survival costs stay constant, then successful invasions are only possible at further increased levels of penetrance (data not shown, but compare figure 5 for the female-specific equivalent): At higher transmission rates, less uninfected females are present in the population and the reproductive advantage of mutant males shrinks accordingly. Therefore, a mutant's reproductive benefits can only offset equal survival costs at higher penetrance levels. Our results indicate that even high costs of survival may be outweighed at intermediate transmission rates, favouring establishment of the mutant allele as well as, on long evolutionary timescales, the eventual loss of *Wolbachia*.

For the sake of completeness, we also investigated the dynamics of a mutant allele that increases CI in males. As expected, such enhancing mutations were not observed to spread in our numerical simulations because mutant males incur higher costs of CI than corresponding wildtypes.

### Female-specific Mutations

Here we consider mutants in the host that either indirectly change the level of *Wolbachia*'s rescue function in the gamete by altering *Wolbachia* transmission rates (e.g. by regulating *Wolbachia*-density during oogenesis or increasing bacterial transmission efficiency) or that directly adjust said rescue function. We then ask whether such mutations can spread in the population. Again, we also introduce a survival cost imposed on mutant females and analyse the two cases in turn. The dynamics are expected to be influenced by the fact that females with repressive mutations will tend to be incompatible with infected males in the population, thus incurring an additional “cost” due to CI.

#### Altering transmission rates

Turelli has shown previously that selection on hosts tends to increase transmission rates in infected females, even in the presence of fecundity costs [21]. Here, we revisit these results with a special focus on the long-term persistence of *Wolbachia*, thereby extending Turelli's work by including in our consideration survival costs incurred by mutant hosts. Figure 3b shows a typical example of a cost-free mutation's dynamics: The original equilibrium between infected and uninfected individuals is left and the prevalence of *Wolbachia* covaries with the mutation's rise in frequency until a new equilibrium between infected and uninfected mutants is reached. At equilibrium, the new “effective” transmission rate is equivalent to (1−*d*)*t*. Therefore, as in the male case, the resulting equilibrium prevalences can be calculated by replacing *t* in equation 10 and 11 in Appendix S1 with *t* ^eff.^ = (1−*d*)*t* (see figure 4b for a graphical example and equations 35 and 36 of the Appendix S1).

In general, whether the mutant allele can invade is influenced by level of CI *l*_CI_, the mutation's penetrance −*d*, transmission rate *t*, and the survival cost *c*. Figures 5a and 5b show the dependence of invasion success on *l*_CI_ and −*d* for different values of *c* and *t*. These results demonstrate that, as expected, cost-free mutations increasing transmission rates can invade and spread through an infected population over nearly the entire parameter space. This is due to the fact that mutant females produce more infected female offspring that can mate compatibly with infected males, which offsets the cost of simultaneously begetting more infected males that are subject to CI in mating with uninfected females.

Prior treatments did not consider the influence of costs to enhancing female mutations. Introducing such costs complicates the matter in that reproductive benefits enjoyed by mutant females must now offset these costs in order for the mutation to spread. As a result, the parameter subspace where the mutation can invade successfully shrinks for elevated survival costs *c* (see figures 5a and 5b). Still, if the mutation does spread, the resulting elevated prevalence levels can be calculated as in the costless case (see above and figure 4b). However, since sex ratios are skewed when *c*>0 (as costs are only incurred by females), the analytical predictions of equations 35 and 36 in Appendix S1 only hold for the overall prevalence of *Wolbachia* but not for the individual equilibria of males and females.

For the sake of completeness, we also investigated the fate of female-specific repressive mutations that lower transmission rates. As expected, these were not observed to spread since mutant females incur the cost of receiving more uninfected daughters that cannot mate compatibly with infected males. Therefore, mutant females are at a fitness disadvantage to corresponding wildtypes that beget more infected offspring – a manifestation of the addiction-character of *Wolbachia*. Moreover, even the potential prior spread of a male-specific repressor only changes the situation qualitatively (i.e. repressive female mutants would now encounter lowered CI-levels) and thus still precludes the subsequent spread of female repressors.

Hypothetical fecundity costs incurred by infected females might be expected to change the general situation, as lowering transmission rates would then harbour the benefit of reducing average fecundity costs since more uninfected offspring are produced. Nevertheless, even in the presence of moderate fecundity costs of up to 10%, such a mutation was never observed to spread during our numerical simulations. This may be due to the fact that fecundity costs decrease the prevalence of *Wolbachia* at equal transmission rates and CI-levels because of the reduced fitness of infected females (see eqs. 13–18 of Appendix S1). Therefore, the infection becomes unstable already at larger critical CI-levels (see eq.19 of Appendix S1), and the critical penetrance levels *e*_crit_ and *d*_crit_ required for loss of *Wolbachia* shrink accordingly. It seems that, as a result, the reduction of fecundity costs in uninfected female offspring does not suffice to offset the increased costs of CI incurred by mutant females.

For similar reasons, we observed little effect of fecundity costs when examining the success of female-specific enhancing mutations that increase transmission in situations with varying fecundity costs. At first glance, we would expect mutant benefits of receiving more infected offspring (that are not subject to CI) to trade-off with fecundity reduction of said infected offspring. However, as the presence of fecundity costs results in a larger proportion of uninfected hosts (see above), this increases the reproductive benefits enjoyed by mutant females, whereas the costs of fecundity reduction remain unaltered. As a result, threshold levels of penetrance above which the mutation may invade and spread are not shifted upwards, as intuition might suggest, but the entire curve is shifted to the right – that is, not higher penetrance thresholds at similar CI-levels, but higher penetrance thresholds at correspondingly higher levels of CI are the result of the introduction of fecundity costs (see figure 2 in Appendix S1 for an illustration).

#### Changing the rescue function directly

Enhancement of *Wolbachia* action in females could also occur if mutant females increase the rescue function directly without affecting transmission rates. In theory such an effect could be achieved if a (hypothetical) *Wolbachia* rescue gene were to be transferred to the host's nucleus. This is similar to the ‘nuclear rescue construct’ of Sinkins and Godfray [24] and has been considered theoretically for cost-free mutations reducing the susceptibility of uninfected ova by Turelli [21], albeit without the focus on potentially resulting infection instabilities and loss of *Wolbachia*. Here, we add costs to the mutations and determine the effects on infection stability.

Generally, the consequences are reminiscent of a male-specific repressive mutation: The mutation induces an autonomous rescue-function in the host (irrespective of whether mutant females are infected with *Wolbachia*) that leads to a decrease in population CI-levels. These may in turn cause the extinction of *Wolbachia* if penetrance levels are above a threshold 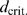. (equivalent to 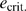 as derived above). If penetrance levels are below this threshold, new equilibria of reduced *Wolbachia* prevalence are attained.

As in the male case, such a mutation always spreads to fixation if it carries no cost for female mutants. However, if mutant females' survival chances are reduced, then penetrance levels need to be above certain thresholds for the mutation to spread (see figure 5c). Interestingly, these thresholds are up to 100% higher than for a male-specific repressive mutation (compare to figure 2). This is due to the fact that mutant males only benefit from a repressive mutation if they are infected and thus would otherwise incur the cost of CI if mated with uninfected females. Mutant females, however, only benefit from enhancing mutations mimicking *Wolbachia*'s rescue function if they are, in fact, uninfected with *Wolbachia* – otherwise their matings would not be subject to CI anyway. These results stress the importance of considering both male- and female-specific CI-reducing mutations and demonstrate that the two scenarios can, in fact, result in qualitatively different dynamics for similar parameter settings.

In contrast, the analogous mutation in females that autonomously lowers the rescue function irrespective of infection status (i.e. an “inverse mimic”) was not successful: Similar to the case of a male-specific enhancing mutation, mutants incur a higher cost of CI due to lowered rescue-levels. Thus, they suffer a fitness disadvantage relative to the wildtype, so that the mutation is not expected to spread (and was not observed to do so in our simulations).

### Non sex-specific mutations

We also examined the fate of a repressive mutation that is equally active in females and in males (with *d* = *e*) so that transmission rates and *Wolbachia*'s modification of sperm are reduced simultaneously. As discussed above, female-specific mutations reducing transmission rates were never observed to spread. Moreover, mutations that simultaneously repress *Wolbachia* manipulation of gametes in males could not spread either. As the results with effects only on females did not differ numerically from those with equal effects on both sexes, it seems that the reproductive costs and benefits of reduced transmission rates are the limiting factor for the spread of any mutation that affects transmission (and thus gametogenesis) also (or only) in females. Note that this would effectively limit the evolution of repressive host modifier alleles as long as their effect – for example on reduction of *Wolbachia* density during gametogenesis – is symmetric in both sexes.

### Sequential Evolution

Hypothetically, several mutations affecting *Wolbachia* action in the host could also arise subsequently within one population (be it through occasional migrational influx or through actual local mutation). Their fate then depends on local values of *l*_CI_, *t*, *e*, *d*, and *c*, as well as on the timing of mutational events:

If a costly male mutant arrives first, spreads, and *Wolbachia* disappears as a result, then future mutations enhancing *Wolbachia* action (whether through increased transmission rate, increased rescue-function, or a ‘nuclear rescue construct’) necessarily are of no effect. However, if the male-specific repressive mutation's penetrance is lower, then *Wolbachia* will persist, albeit at lower prevalence as a result of the mutation's spread. As a consequence, invasion conditions for female-specific enhancing mutations are rendered more restrictive as the spread of the male mutation lowers the effective level of CI to (1−*e*)*l*_CI_ (compare figure 5 where threshold values of −*d* rise with lower *l*_CI_). In contrast, conditions for the subsequent spread of additional male-specific repressive modifiers are eased (i.e. threshold levels of *e* fall with lower *l*_CI_).

**Figure 6:**
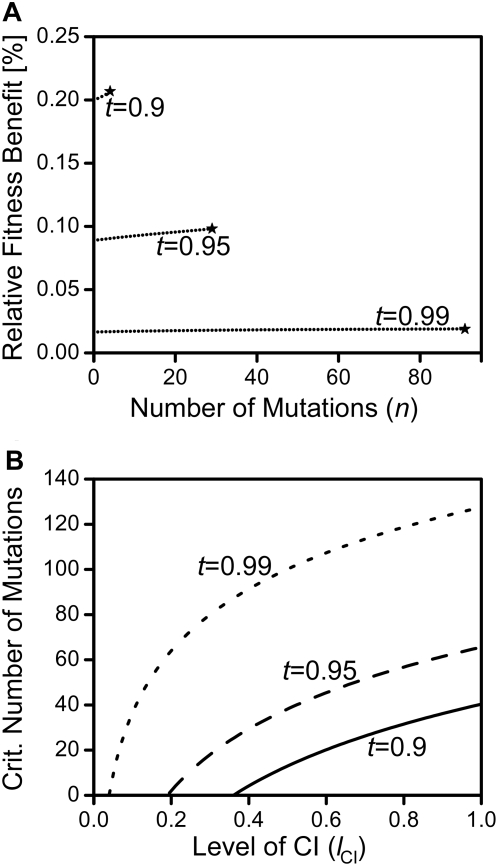
Cascades of male-specific repressive mutations and loss of *Wolbachia*. Shown in 6a are the increasing fitness advantages during a cascade of male-specific repressive mutations of equally small effect. The percentage fitness benefits of an additional mutation relative to the predominating genotype are plotted against the currently fixed number of *n* mutations. Fitness benefits are approximated using eqs. 27–28 of Appendix S1. Each dot represents one mutational step in the cascade; stars indicate the final stop of loss of *Wolbachia* where cumulative effects reach the threshold penetrance *e*_thr._. Parameters are *e* = 0.025 and *l*_CI_ = 0.4 with transmission rates varied as indicated. Shown in 6b are the critical numbers of male-specific repressive mutations *n*_crit._ of equal effect that are necessary to drive *Wolbachia* to extinction in such a cascade of mutations. These thresholds were calculated analytically using eq.37 of Appendix S1 and are plotted as a function of *l*_CI_ with *t* varied as indicated and *e* = 0.025. Other parameters for both graphs are *c* = 0 and *f* = 0.

To explore these predictions further, we examined cascades of mutations of equally small effect and found that these can lead to gradual meltdown of CI and long-term frequency decline and loss of *Wolbachia*.We know from our analyses that cost-free male-specific repressors will spread to fixation for all penetrance levels, thereby driving the earlier predominating wildtype to extinction. Moreover, we can calculate the “effective” CI-level that Wolbachia are exposed to after fixation of a host-modifier. As a result, we can approximate the fitness benefits enjoyed by a new mutation that we introduce after a previous male-specific modifier has reached fixation (see eq.38 of Appendix S1). For the cascade of mutations, we repeatedly calculate the fitness benefits of two such subsequent mutations until the mutations' cumulative effects have reduced CI-levels to *l*_crit._ and *Wolbachia* goes to extinction. The results show that the benefit enjoyed by subsequent male-specific repressors grows with the number of preceding mutations of similar effect (see. figure 6a). As predicted, successive mutations act synergistically and ease their reciprocal spread until the final stop of *Wolbachia*-extinction is reached. Moreover, we can use infection stability to calculate analytically the critical number of repressive mutations of equal effect that is necessary to drive *Wolbachia* to extinction (see eq.37 of Appendix S1). Figure 6b shows these critical numbers to vary greatly depending on the mutation's penetrance as well transmission rates and CI-levels, but for certain parameter values (e.g. *t* = 0.9, *e* = 2.5%) few consecutive mutations suffice to reach the cascade's final stop.

Female enhancers that increase *Wolbachia's* rescue function also lower effective CI-levels and are thus expected to produce qualitatively similar results as male repressors: Either *Wolbachia* disappears (if *d*≥*d*_crit._) and subsequent male-specific repressive mutations are of no relevance, or *Wolbachia* persists at reduced prevalence levels so that conditions for subsequent male-specific repressive mutations are eased due to lowered CI-levels. As noted before, prior fixation of CI-reducing mutations in males or females does not produce conditions conducive to the spread of female specific repressors lowering transmission rates, even in the presence of fecundity costs.

Conversely, if a female-specific enhancing mutation that increases transmission rates arises first and is able to spread, then elevated effective transmission rates will restrict conditions under which costly male mutants may invade (since threshold values of *e* rise with higher *t*). Moreover, such preceding female-specific mutations with a penetrance of 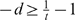 would raise local transmission rates to 1 so that subsequent male-specific repressive mutations cannot spread since they require imperfect transmission. However, perfect transmission is only reached in the absence of fecundity costs. If fecundity costs are present (i.e. *f*>0), then effective transmission rates remain below 1 so that subsequent spread of male-specific repressive mutations is still possible. Furthermore, even under complete transmission and without female fecundity costs, male repressors are expected to invade when there is a male fecundity or survival cost to being infected (which we did not explicitly consider in our model). This is because mutant males are then equally compatible with females whether they have the infection or not, and loss of *Wolbachia* increases fitness. In turn, this would lead to either reduced equilibrium levels or elimination of *Wolbachia*, as before. Moreover, the presence of fecundity costs generally leads to an increase in the critical CI-level below which the infection becomes unstable and *Wolbachia* disappears (see above and eq.19 of Appendix S1). As a result, critical penetrance levels *e*_crit._ shrink accordingly, so that the cumulative evolution of male-specific repressors can lead to quicker loss of *Wolbachia*.

Note that, although not explicitly considered in our study, there also is selection on *Wolbachia* to counter host adaptation, e.g. by increasing transmission rates or increasing CI-levels (as modeled in ref. 21). Such *Wolbachia* adaptations could result in very high symbiont-mediated transmission rates that, in turn, would weaken selection on hosts for reduced sperm modification. However, while perfect transmission has indeed been recorded under laboratory conditions (e.g. [34]), earlier studies suggest that transmission rates can be lower in the field [35]. Also, as mentioned previously, even if complete transmission occurs, selection for male repressors of *Wolbachia* will be favored in the infection imposes a survival or fertility cost in males.

In summary, the outcome of selection can be the consequence of two competing processes – selection for enhanced transmission through females and selection for reduced modification in males, where consecutive mutations work synergistically within each class (that is, they ease the spread of future mutations of similar effect) and antagonistically between classes. While individual outcomes naturally depend on the specific costs and effects of the two classes of mutation, we generally expect cascades of male-specific repressors to outweigh the effect of female-specific enhancers on long evolutionary time scales. This is motivated by the fact that the presence of fecundity costs discourages the emergence of host-mediated perfect transmission. Still, even if transmission is complete, male-specific repressors would be selectively favored if Wolbachia impose a fertilitiy or survival cost on infected males. As a result, we expect this sequential sexually antagonistic process to lead to long-term loss of *Wolbachia*.

## Discussion

*Wolbachia* are among the most widespread and abundant infections known. They are inherited maternally within species and also move laterally between host taxa. The bacteria spread within host species due to the reproductive alterations they induce, such as cytoplasmic incompatibility, feminization, parthenogenesis, or male-killing. Phylogenetic comparisons of hosts and *Wolbachia* indicate that these bacteria do not persist over long time periods within most arthropod taxa [1], [6], [12]. That is, rarely does one find concordant phylogenies of the bacteria and their hosts. Therefore, the infections are relatively quickly lost on evolutionary time scales, such that they infrequently persist within a taxon across speciation events. However, in the case of CI bacteria, infection loss would appear to be difficult because elimination of the infection in females would lead to “sterility” of these females in crosses with infected males, due to CI. This has presented a paradox – why and how are *Wolbachia* infections lost in species.

Our study is an attempt to investigate this question. The results show that sex-specific host repressors of *Wolbachia* in males can readily spread, even at cost to mutant males, and either result in elimination of *Wolbachia* infections or reduced infection levels within a population. Conversely, enhancers of *Wolbachia* transmission in females are favoured under a broad range of conditions, because they enhance the probability of female eggs being compatible with sperm from infected males. Hence selection for host modifiers works antagonistically in the two sexes. The outcome of these processes may be complex, although in general, we expect that successive selection for male repressors of *Wolbachia* can lead to long-term elimination of infections.

There is some empirical support for the notion that male-specific reduction of CI modification occurs. In *Drosophila melanogaster*, *Wolbachia* are effective at inducing CI only in young males, and the infection level declines and can be lost in testes over time [30]. In the cricket species *Gryllus firmus* and *G. pennsylvaticus*, *Wolbachia* are absent from testes, despite the presence of somatic infections [36]. These results may be consistent with selection for sex specific reduction or exclusion of *Wolbachia* from testes. Moreover, introgression experiments demonstrate an effect of host genetic background on level of CI and thus the apparent coevolution of host and reproductive parasite regarding regulation of *Wolbachia* action [30].

Host-induced changes not in the density of *Wolbachia* but in the efficiency of their action also seem to occur: In the parasitoid wasp *Nasonia vitripennis*, complete CI occurs despite very low *Wolbachia*-density in all tissues including the gonads – even if *Wolbachia* is present not in the spermatocysts but only in the adjacent sheet cells [37]. When compared to the situation in *Drosophila* where *Wolbachia*-placement in sheet cells is not sufficient to cause CI [30], these findings suggest host-parasite interactions that alter *Wolbachia*'s efficiency.

The potential role of host suppressor genes has repeatedly been mentioned in the literature (e.g. Stouthamer [38] with regard to *Wolbachia*-induced parthenogenesis) and there is empirical evidence for host-suppression of male-killing as well as parthenogenesis-inducing *Wolbachia* [39]–[42]. Earlier theoretical analyses [21] found host selection to favor increased transmission rates as well as increased compatibility between infected males and uninfected females. However, these treatments did not consider costs to such modifiers, and therefore expected uninfected females from polymorphic populations to generally evolve resistance to incompatibility [21]. Mathematically, the consequences of a mutant female's reduced susceptibility are similar to those of a mutant's males reduced sperm-modification: both lower the effective level of CI. However, our study shows that male-specific repressive mutations lowering CI will spread for a wider range of conditions than will female-specific mutations lowering CI (i.e. a ‘nuclear rescue construct’, sensu Sinkins and Godfray[24]). This difference is due to CI-reduction differentially affecting both sexes regarding their infection status – in mutant females uninfected individuals benefit more, whereas among mutant males infected individuals enjoy greater benefits.

Our study further demonstrates that even complete suppressors of *Wolbachia*-growth (i.e. *e* = 1) can increase within populations at a stable infection frequency equilibrium when they are male-specific. This contrasts with some earlier predictions that did not take into account the possibility of sex-specific host modification of *Wolbachia* action [21], specifically repression of *Wolbachia* in male gonads. Later modelling work that did include sex-specific host modifications and focused on the special case of complete elimination of CI found such mutations to spread easily [22]. Similarly, a theoretical study concentrating on the effect of CI-types in haplodiploids demonstrates the spread of CI-reducing host modifiers with moderate to large effects [23]. However, both these studies do not investigate systematically the fate of mutations of varying effect size, fitness costs to the repressors, and the mutations' consequences for the long-term persistence or extinction of *Wolbachia*. Further verbal arguments of sex-specific “resistance” expected nuclear genes increasing transmission rates in females to spread if *Wolbachia*-prevalence is higher than 50%, while male resistance was expected to spread if less than 100% are infected [14]. Our formal model then confirms these predictions but also shows that male suppressors may spread at initial infection frequencies of 100% (i.e. for *l*_CI_ = 1 and *t*<1, where uninfected zygotes are produced but fail to develop due to perfect CI).

Generally, our study systematically extends the scope of possible mutations to the entire range of host modifications (i.e. from 0% to 100%). By explicitly considering the consequences for infection instability, we were able to deduce analytically those critical penetrance levels above which the spread of a modifier leads to loss of *Wolbachia* . Additionally, host modifications with small effect on, for example, *Wolbachia* gonadal density, seem to be biologically more plausible than the direct elimination of CI altogether. Overall, we show that spread of a male repressor can lead to loss of the *Wolbachia* under a broad range of clearly-specifiable conditions. Moreover, spread of sub-threshold male repressors eases invasion conditions for subsequent mutations of similar kind, so that *Wolbachia* may be lost in a step-wise manner. As a result, increase of such sex-specific repressors may be one process explaining why CI-*Wolbachia* do not persist within host taxa on evolutionary timescales.

Despite *Wolbachia*'s lifestyle as an intracellular parasite, the expected phylogenetic congruence between hosts and *Wolbachia* is absent [1], [6]–[8], [12], [15], [17]. Our model suggests these data to be interpreted as the result of repeated loss of CI-inducing *Wolbachia* through the spread of host modifiers and subsequent reinfection by horizontal transfer. Earlier studies point out that invasion of non-modifying *Wolbachia* into infected populations could also account for the subsequent loss of *Wolbachia* [25]. However, this explanation requires a direct cost of *Wolbachia* to be imposed on infected females in order for *Wolbachia* to be lost through selection. Within our model, however, a hypothetical moderate fecundity cost *f* that lowers infected females' fecundity levels to 1−*f* had no effect on invasion success. Assuming such direct costs of *Wolbachia* is thus unnecessary for *Wolbachia* to be lost from the population through the mechanisms proposed by our model. This stands in contrast to previous theoretical studies of suppression of CI-inducing *Wolbachia* [22], [23] that did not separately consider scenarios without fecundity costs incurred by infected females.

Empirical studies have shown two types of CI to exist in haplodiploid insects where CI-affected offspring either die (female mortality, FM) or develop as males (male development, MD; [43]). Previous studies show that, in contrast to the typical FM-CI, the MD-type selects for females to decrease transmission rates [23]. While we did not explicitly consider this CI-type in our own modelling, this fact nonetheless substantiates the possibility of *Wolbachia* loss. Moreover, empirical results suggest that dosage effects of *Wolbachia*-induced damage to paternal DNA may account for these differential CI-types: In simplified terms, greater damage leads to haploid embryos (and thus the MD-type in haplodiploids) whereas smaller damage allows for some paternal chromosomes to survive the first mitosis, which results in aneuploid embryos and failure of development (the FM-type; [44], [45]). As a result, host-mediated changes in bacterial density might lead to changes in CI-type, as observed by Breeuwer and Werren [44]. Thus, the host modifiers examined in our study – especially those that have small effects on bacterial action and lead to reduced prevalence, not extinction of *Wolbachia* – may provide a host-mediated pathway between different CI-types. Note that, however, even though the MD-type is more prone to host modification than the FM-type (see above and [23]), the type of CI will only have a quantitative influence on the evolution of *Wolbachia*-suppression, not a qualitative one: It may increase fixation times for an allele reducing CI-levels in FM- relative to MD-populations, but it does not affect the evolutionary potential of such modifier alleles on long evolutionary time scales.

Finally, given our results of the easily-observable loss of *Wolbachia*-infection, the details of the host-symbiont system under study here can be regarded as not stable but in fact circular: Any uninfected and unregulatory host-population could be infected by CI-*Wolbachia* (given high enough values of *t* and *l*_CI_), thus leading to spread of the infection. Subsequent selection on the host for sex-specific repressors would lead to eventual loss of the *Wolbachia*. Once the repressors declined due to mutation or selection against a costly repressor in the absence of *Wolbachia*, the species would be vulnerable to reinfection by CI inducing *Wolbachia*. Thus infections could be cyclical within a host clade over evolutionary time. In the spirit of previous work [46], infection with CI-inducing *Wolbachia* does not seem to be a veritable “final stop” for any insect host population.

## Supporting Information

Appendix S1 Appendix S1 contains mathematical formulae and derivations as well as additional figures.
(0.45 MB DOC)
