# Supplementary material for: Life and Death of an Influential Passenger: Wolbachia and the Evolution of CI-Modifiers by Their Hosts
Source: PLoS One. 2009 Feb 11;4(2):e4425. doi: 10.1371/journal.pone.0004425 (PMC2635967; doi:10.1371/journal.pone.0004425)
Supplement: Appendix S1 — contains mathematical formulae and derivations as well as additional figures. (0.45 MB DOC) [file pone.0004425.s001.doc]

Appendix S1

Model formalization

Here we give a detailed description of the applied mathematical model including a definition of all variables and parameters. In order to study the evolutionary fate of a rare repressive or enhancing mutant, we derive a haploid sexual model of a single, panmictic host population. Individuals are sufficiently described by three parameters: the nuclear suppressor locus R with the two alleles R_1_ (mutant) and R_0_ (wild-type), the cytoplasmic infection status with *Wol­ba­chia* with the options infected or non-infected, and of course their sex. Therefore, we derive difference equations describing the frequency changes of the eight phenotypic classes of individuals identified by this set of three binary parameters. The phenotypic classes are thereby represented by *x_p,q,r_* where the subscripts stand for the three parameters sex, cytotype, and genotype. For example, the frequency of non-infected mutant males is thus given by *x*_1,0,1_ while *x*_0,1,0_ denotes the frequency of infected wild-type females.

To obtain an expression for the frequency of all offspring classes in the next generation as a function of the parental phenotypic class distribution ($x_{\text{p,q,r}}^{\text{'}}=f(x_{0,\beta,\gamma},x_{1,\delta,\varepsilon})$), we must take into account the effects of four separate processes: The possibly imperfect transmission of *Wol­ba­chia*, the harmful effects of *Wol­ba­chia*-induced cytoplasmic incompatibility on the offspring, the fecundity cost carried by *Wol­ba­chia*-infected females, and the survival costs suffered by mutant individuals. At the same time, inheritance at the modifier locus follows simple Mendelian rules. We will now first develop weighting factors that describe the effects mentioned above and then derive the desired system of difference equations.

Transmission:

Relevant to the model-context, the production of offspring begins with the possible transmission of *Wol­ba­chia* from the mother to the gamete. Thus, we define a weighting factor $T_{\beta,q}$ describing the maternal transmission of *Wol­ba­chia* to the offspring that only depends on *β* and *q*, the infection status of the mother and of the offspring, respectively. Here *t* refers to transmission rate, i.e. the probability that gametes produced by an infected mother also are infected.

$\text{T}_{\beta,\text{q}}=1-\text{q}+\beta\text{t}\left(2\text{q}-1\right)$ (1)

However, taking a female-specific mutation into account, we assume that the acquired ability to enhance or repress *Wol­ba­chia* transmission in the gonads leads to exclusion of *Wol­ba­chia* from any gamete according to the mutation’s penetrance level *d*. Hence, the effective transmission rate is altered and $T_{\beta,q}$ also comes to depend on *γ*, the genotype of the mother:

$\text{T}_{\beta,\text{q},\gamma}=\beta\text{q}\left(1-\gamma\text{d}\right)\text{t}+\left(1-\text{q}\right)\left(1-\beta\left(1-\gamma\text{d}\right)\text{t}\right)$ (2)

In order to simulate those female-specific cases where *Wol­ba­chia*’s rescue function was influenced directly without altering transmission rates, we reverted to the earlier formula where $T_{\beta,\text{q}}$ is independent of the mother’s genotype *γ*.

Cytoplasmic incompatibility:

The second step in offspring-generation to be incorporated into the model is the harmful effects of cytoplasmic incompatibility induced by *Wol­ba­chia* in the offspring. Consequently, we define a weighting-factor $\text{L}_{\delta,\text{q}}$ that describes these effects and depends on *q*, the infection status of the maternal gamete after transmission, and on *δ*, the infection status of the father. We take *l*_CI_ to represent the level of CI, i.e. the probability that uninfected maternal gametes fertilized by infected fathers do not develop.

$\text{L}_{\delta,\text{q}}=1-\delta\left(1-\text{q}\right)\text{l}_{CI}$ (3)

However, if we take into account male-specific repressive mutations that enable the father to repress *Wol­ba­chia*’s manipulation of spermatozoids according to the mutation’s penetrance *e*, then the term for $L_{\delta,q}$ also comes to depend on *ε*, the genotype of the father:

$L_{\delta,\text{q},\varepsilon}=1-\delta\left(1-\text{q}\right)\left(1-\varepsilon\cdot\text{e}\right)\text{l}_{CI}$ (4)

Moreover, if we take into account a male-specific mutation enhancing *Wol­ba­chia*’s rescue function, we need to let the mutation’s penetrance take on negative values so that “effective” CI-levels rise. Also, for the case of a female-specific mutation directly affecting *Wol­ba­chia*’s rescue function, we need to replace the male-specific penetrance *e* with the female one *d* as well as the father’s genotype *ε* with that of the mother *γ*. However, as enhancing the rescue-function will decrease CI-levels while repression elevates CI-levels, the denotation of the penetrance level *d* changes: Positive values now translate to enhancement while negative values denote repression of *Wol­ba­chia* action.

Mendelian inheritance:

We then define a weighting-factor $\text{M}_{\gamma,\varepsilon,\text{r}}$ to describe the next step in offspring-production, the Mendelian inheritance of the modifier locus R. This factor depends on the genotype of the parents *γ* and *ε*, as well as on the offspring-genotype in question *r*:

$\text{M}_{\gamma,\varepsilon,\text{r}}=1-\frac{1}{2}\left(\varepsilon+\gamma\right)-\text{r}\left(1-\gamma-\varepsilon\right)$ (5)

Fecundity costs:

We now define a weighting factor $\text{F}_{\beta}$ to integrate possible fecundity costs imposed on *Wol­ba­chia*-infected females into the model. This factor only depends on *β*, the infection status of the mother. Here *f* stands for fecundity costs: an infected mother is assumed to have (1−*f*) less offspring than an uninfected one.

$\text{F}_{\beta}=1-\beta\cdot\text{f}$ (6)

Cost of suppression:

Mutants enabled to repress or enhance *Wol­ba­chia* action also carry a cost: They are assumed to have a (1−*c*) smaller chance of survival, where *c* denotes the actual cost of the mutation. Accordingly, we define a weighting factor $\text{C}_{\text{p,r}}$ that depends on *p*, the sex of the offspring and on their genotype *r*:

$\text{C}_{\text{p,r}}=1-\text{p}\cdot\text{r}\cdot\text{c}$ (7)

This weighting factor can be structurally changed by altering the dependence on *p*, the sex of the class in question, in order to vary between one or both sexes carrying the costs of density-regulation.

Difference equations

We then construct the difference equations describing the frequency changes of all eight phenotypic classes from generation to generation. Building on standard replicator dynamics, we sum over all possible parental class combinations for each offspring class and multiply with the appropriate weighting factors defined above. Since each class-describing parameter is of binary nature, every sum merely contains two terms and hence goes from zero to one only.

(8)

In these equations, the average fitness is the sum over all possible of the above right-hand side terms:

(9)

We are now able to simulate the desired fate of a mutant in a single population by numerically iterating these coupled difference equations. As specified in the text, we can modify the model by changing the parameters *t* (to manipulate accuracy of *Wol­ba­chia*-transmission), *l*_CI_ (to change cytoplasmic incompatibility-level), and *f* (to specify the fecundity cost inflicted by *Wol­ba­chia*) according to the desired *Wol­ba­chia*-dynamics. Consequently, we are able to implement any specific expression-level of mutation by adjusting the respective penetrance levels *d* and *e* as well as the survival cost *c* incurred by mutants.

Analytical results

Without mutants

The reduced system without the mutated classes (i.e. with *x_p,q_,*_1_ = 0) can be solved analytically to reveal three quartets of fixed-points that correspond to stable extinction of *Wol­ba­chia*, the instable invasion threshold of *Wol­ba­chia*-spread, and stable *Wol­ba­chia*-persistence. The results for stable *Wol­ba­chia*-persistence are:

$\text{x}_{0,0,0}=\text{x}_{1,0,0}=\frac{1}{2}\left(1-\frac{1}{2\text{t}}-\frac{\sqrt{\text{l}_{CI}-4\text{t}\left(1-\text{t}\right)}}{2\sqrt{\text{l}_{CI}}\text{t}}\right)$ (10)

$\text{x}_{0,1,0}=\text{x}_{1,1,0}=\frac{\sqrt{\text{l}_{CI}}+\sqrt{\text{l}_{CI}-4\text{t}\left(1-\text{t}\right)}}{4\sqrt{\text{l}_{CI}}\text{t}}$ (11)

Thus, *Wol­ba­chia* only persists in the population for

$\text{l}_{CI}\geq4\text{t}\left(1-\text{t}\right)$ (12)

This value was used as a lower bound for *l*_CI_ in the numerical simulations while the corresponding steady-state prevalence levels were used as starting conditions.

With infected females bearing fecundity costs

Adding fecundity costs to the reduced system without the mutated classes (i.e. with *x*_p,q,1_ = 0 and *f*≠0) reveals a new system with three quartets of fixed points:

$x_{0,0,0}^{(1)}=x_{1,0,0}^{(1)}=\frac{1}{2}$ (13)

$x_{0,1,0}^{(1)}=x_{1,1,0}^{(1)}=0$ (14)

$\text{x}_{0,0,0}^{(2)}=\text{x}_{1,0,0}^{(2)}=\frac{1}{2}+\frac{\text{f}+\text{l}_{CI}-\sqrt{\left(\text{f}+\text{l}_{CI}\right)^{2}+4\text{l}_{CI}\left(\text{f}\left(\text{t}-1\right)-\text{t}\right)\left(1+\text{t}\left(\text{f}-1\right)\right)}}{4\text{l}_{CI}\left(\text{f}\left(\text{t}-1\right)-\text{t}\right)}$ (15)

$\text{x}_{0,1,0}^{(2)}=\text{x}_{1,1,0}^{(2)}=-\frac{\text{f}+\text{l}_{CI}-\sqrt{\left(\text{f}+\text{l}_{CI}\right)^{2}+4\text{l}_{CI}\left(\text{f}\left(\text{t}-1\right)-\text{t}\right)\left(1+\text{t}\left(\text{f}-1\right)\right)}}{4\text{l}_{CI}\left(\text{f}\left(\text{t}-1\right)-\text{t}\right)}$ (16)

$\text{x}_{0,0,0}^{(3)}=\text{x}_{1,0,0}^{(3)}=\frac{1}{2}+\frac{\text{f}+\text{l}_{CI}+\sqrt{\left(\text{f}+\text{l}_{CI}\right)^{2}+4\text{l}_{CI}\left(\text{f}\left(\text{t}-1\right)-\text{t}\right)\left(1+\text{t}\left(\text{f}-1\right)\right)}}{4\text{l}_{CI}\left(\text{f}\left(\text{t}-1\right)-\text{t}\right)}$ (17)

$\text{x}_{0,1,0}^{(3)}=\text{x}_{1,1,0}^{(3)}=-\frac{\text{f}+\text{l}_{CI}+\sqrt{\left(\text{f}+\text{l}_{CI}\right)^{2}+4\text{l}_{CI}\left(\text{f}\left(\text{t}-1\right)-\text{t}\right)\left(1+\text{t}\left(\text{f}-1\right)\right)}}{4\text{l}_{CI}\left(\text{f}\left(\text{t}-1\right)-\text{t}\right)}$ (18)

Here, quartet (1) represents the loss of *Wol­ba­chia*, quartet (2) the invasion threshold of *Wol­ba­chia*-infection, and quartet (3) the steady-state equilibria of successful infections. Thus, *Wol­ba­chia*-infections only persist for conditions where fixed- point-quartets (2) and (3) are real and non-negative. The root present in these formulae is real if

$\text{l}_{CI}\geq2\text{t}\left(1-\text{t}\right)\left(1-\text{f}\left(2-\text{f}\right)\right)+\text{f}+\sqrt{\left(2\text{t}\left(1-\text{t}\right)\left(1-\text{f}\left(2-\text{f}\right)\right)\right)\left(2\text{t}\left(1-\text{t}\right)\left(1-\text{f}\left(2-\text{f}\right)\right)+2\text{f}\right)}$ (19)

$\vee\text{ l}_{CI}\geq2\text{t}\left(1-\text{t}\right)\left(1-\text{f}\left(2-\text{f}\right)\right)+\text{f}-\sqrt{\left(2\text{t}\left(1-\text{t}\right)\left(1-\text{f}\left(2-\text{f}\right)\right)\right)\left(2\text{t}\left(1-\text{t}\right)\left(1-\text{f}\left(2-\text{f}\right)\right)+2\text{f}\right)}$ (20)

The root present in these formulae is real for *f* ≤ 1 and *t* ≤ 1 (which is always the case). However, if *l*_CI_ suffices the condition of equation 20, then fixed-point-quartets (2) and (3) contain negative terms. Thus, the condition for *l*_CI_ presented by equation 19 represents the threshold of CI-level below which *Wol­ba­chia*-infections cannot persist. Therefore, this threshold was used as a lower bound for *l*_CI_ in the numerical simulations with fecundity costs (*f* > 1), while the analytically-determined steady-states were used as starting conditions.

With mutants bearing survival costs

In order to approximate the threshold value *e*_thr_ above which a costly mutation may spread, we seek the value for *e* where the fitness advantage enjoyed by mutants equals their survival costs. With subscripts indicating sex and superscripts indicating genotypes, this can be formalised as:

$\frac{\frac{1}{2}w_{\text{m}}^{\text{Mut}}+\frac{1}{2}w_{\text{f}}^{\text{Mut}}}{\bar{w}} \left(1-c\right)=1$ (21)

Since the mutation is initially very rare, we assume $\bar{w}=w^{\text{WT}}$. In order to estimate the respective fitness values, we first consider the effect of CI on the reproductive values of all possible offspring classes. These are calculated by estimating the probability of all offspring-matings multiplied by the respective costs incurred through CI. Hence, these reproductive values in turn depend on *x*_W_, the frequency of individuals infected with *Wol­ba­chia*. With nomenclature as above and superscripts also indicating infection status (I for infected or 0 for uninfected), this gives:

$$RV_{\text{m}}^{0,\text{Mut}}=RV_{\text{m}}^{0,\text{WT}}=1$$

$$RV_{\text{f}}^{0,\text{Mut}}=RV_{\text{f}}^{0,\text{WT}}=1-l_{\text{CI}} x_{W}$$

$RV_{\text{m}}^{\text{I},\text{WT}}=1-l_{\text{CI}} \left(1-t x_{W}\right)$ (22)

$$RV_{\text{m}}^{\text{I},\text{Mut}}=1-\left(1-e\right) l_{\text{CI}} \left(1-t x_{W}\right)$$

$$RV_{\text{f}}^{\text{I},\text{WT}}=RV_{\text{f}}^{\text{I},\text{Mut}}=1-l_{\text{CI}} \left(1-t\right) \left(1-x_{W}\right)$$

We then calculate the proportion of each offspring class produced by wildtypes, mutant males and mutant females, respectively. For wildtypes (and with nomenclature as above), this gives:

$p_{\text{m}}^{\text{I},\text{WT}}=p_{\text{f}}^{\text{I},\text{WT}}=\frac{1}{2} t x_{W}$ (23)

$p_{\text{m}}^{0,\text{WT}}=p_{\text{f}}^{0,\text{WT}}=\frac{1}{2} \left(1-l_{\text{CI}} x_{W}\right) \left(1-t x_{W}\right)$ (24)

For mutant males (hatted) and mutant females (dotted), this gives:

$\hat{p}_{\text{m}}^{\text{I},\text{WT}}=\hat{p}_{\text{f}}^{\text{I},\text{WT}}=\hat{p}_{\text{m}}^{\text{I},\text{Mut}}=\hat{p}_{\text{f}}^{\text{I},\text{Mut}}=\frac{1}{4} t x_{W}$ (25)

$\hat{p}_{\text{m}}^{0,\text{WT}}=\hat{p}_{\text{f}}^{0,\text{WT}}=\hat{p}_{\text{m}}^{0,\text{Mut}}=\hat{p}_{\text{f}}^{0,\text{Mut}}=\frac{1}{4} \left(1-\left(1-e\right)l_{\text{CI}} x_{W}\right) \left(1-t x_{W}\right)$ (26)

(27)

(28)

Summing over the products of each class’ proportion and reproductive value then gives total fitness. This is exemplified for the wildtype here; expressions for mutant male and mutant female fitness values are derived in a similar spirit:

$w^{WT}=p_{\text{m}}^{\text{I},\text{WT}} \left(RV_{\text{m}}^{\text{I},\text{WT}}+RV_{\text{f}}^{\text{I},\text{WT}}\right)+p_{\text{m}}^{0,\text{WT}} \left(RV_{\text{m}}^{0,\text{WT}}+RV_{\text{f}}^{0,\text{WT}}\right)$ (29)

Finally, the results are substituted into equation 21 which is solved for *e* to reveal the threshold efficiency of density regulation:

$e_{\text{thr}}=\frac{2c\left(2+l_{\text{CI}}^{2}x_{W}^{2}\left(1-tx_{W}\right)-l_{\text{CI}}x_{W}\left(3-t^{2}+t\left(2-4x_{W}\right)\right)\right)}{l_{\text{CI}}x_{W}\left(1-c\right)\left(2+t-l_{\text{CI}}x_{W}\right)\left(1-tx_{W}\right)}$ (30)

The frequency of infected individuals *x*_W_ may be calculated from the equilibrium frequencies given by equation 11 using *x*_W_ = *x*_1,0,1_ + *x*_1,1,0_. The resulting threshold levels of *e* can then be plotted as a function of *l*_CI_ with constant *t* and *c* (see figure 2 in the main text). As projection of the population dynamics only one generation into the future fails to take into account the inclusive fitness benefit enjoyed by mutant females, the accuracy of estimation (especially for low values of *l*_CI_) may be increased by including another future generation in the approximation algorithm derived above. This is illustrated in figure 1 of this appendix.

With mutants bearing no survival costs

The net benefits of mutant males and females can be calculated by subtracting the wildtype fitness from their respective fitness values, all as derived in equation 29:

$\Delta w_{\text{m}}=w_{\text{m}}^{\text{Mut}}-w^{\text{WT}}=\frac{1}{4} e l_{\text{CI}} x_{W}\left(4+t-2 l_{\text{CI}} x_{W}\right) \left(1-t x_{W}\right)$ (31)

$\Delta w_{\text{f}}=w_{\text{f}}^{\text{Mut}}-w^{\text{WT}}=\frac{1}{4} e l_{\text{CI}} x_{W}\left(1-t x_{W}\right)$ (32)

These differences are positive if transmission is imperfect (*t*<1) and zero for perfect transmission (*t*=1). Thus, a mutant without survival costs is expected to spread for any *e*>0 given imperfect transmission.

Mutationally altered equilibria

After the fixation of a male-specific repressive mutation with $e<1-\frac{4t\left(1-t\right)}{l_{CI}}$, a new equilibrium of infected and non-infected mutants is reached. Replacing *l*_CI_ in eqs. 10 & 11 by $l_{CI}^{eff.}=\left(1-e\right)l_{CI}$ reveals these fixed points (for *c*=0) to be:

$x_{0,0,1}=x_{1,0,1}=\frac{1}{2}\left(1-\frac{1}{2t}-\frac{\sqrt{\left(1-e\right)l_{\text{CI}}-4t\left(1-t\right)}}{2\sqrt{\left(1-e\right)l_{\text{CI}}}t}\right)$ (33)

$x_{0,1,1}=x_{1,1,1}=\frac{\sqrt{\left(1-e\right)l_{\text{CI}}}+\sqrt{\left(1-e\right)l_{\text{CI}}-4t\left(1-t\right)}}{4\sqrt{\left(1-e\right)l_{\text{CI}}}t}$ (34)

In the same spirit, after the fixation of a female-specific enhancing mutation increasing transmission rates with $-d\leq\frac{1}{t}-1$, a new equilibrium of infected and non-infected mutants is reached. Replacing *t* in eqs. 10 & 11 by *t*^eff^. = (1-*d*)*t* reveals these fixed points (for *c* = 0) to be:

$x_{0,0,0}=x_{1,0,0}=\frac{1}{2}\left(1-\frac{1}{2\left(1-d\right)t}-\frac{\sqrt{l_{\text{CI}}-4\left(1-d\right)t\left(1-\left(1-d\right)t\right)}}{2\sqrt{l_{\text{CI}}}\left(1-d\right)t}\right)$ (35)

$x_{0,1,0}=x_{1,1,0}=\frac{\sqrt{l_{\text{CI}}}+\sqrt{l_{\text{CI}}-4\left(1-d\right)t\left(1-\left(1-d\right)t\right)}}{4\sqrt{l_{\text{CI}}}\left(1-d\right)t}$ (36)

Sequential evolution

Regarding a cascade of mutations of equal effect, we can approximate the relative fitness benefits of consecutive mutations. For male repressors, a successful sub-threshold mutation decreases CI-levels to (1-*e*)*l*_CI_, so that successional fixation of *n* mutations of equal penetrance *e* will cumulatively lower population CI-levels to $\left(1-e\right)^{n}l_{CI}$. Setting this equal to 4*t*(1-*t*) lets us calculate the critical number of subsequent mutations *n*_crit.´_of equal penetrance *e* that is necessary to drive *Wol­ba­chia* to extinction:

$n_{\text{crit}.}=\frac{ln\left(4t\right)+ln\left(1-t\right)-ln l_{\text{CI}}}{ln\left(1-e\right)}$ (37)

Moreover, we can calculate the fitness benefit of the next mutation *n*+1 relative to the currently predominating genotype of *n* accumulated mutations for each step of the cascade of mutations until *Wol­ba­chia* is lost. With superscripts in brackets indicating the number of mutations (where *n*=0 would represent the wildtype), this amounts to:

$\frac{w_{\text{m}}^{\text{Mut}\left(n+1\right)}+w_{\text{f}}^{\text{Mut}\left(n+1\right)}}{2\bar{w}^{\left(n\right)}}$ (38)

Here, we make use of the fact that we can represent the predominating genotype of *n* accumulated mutations as the original wildtype but with CI-levels reduced to $\left(1-e\right)^{n}l_{CI}$ (see above). Using the fitness approximations derived above (eq.21–29), this approach reveals that fitness benefits rise with increasing number of subsequent mutations (see figures 6a and 6b of the main text). Prior CI-reduction eases conditions for the spread of future male-specific repressors, and male repressors thus act synergistically until *Wol­ba­chia* is driven to extinction.

Appendix Figures


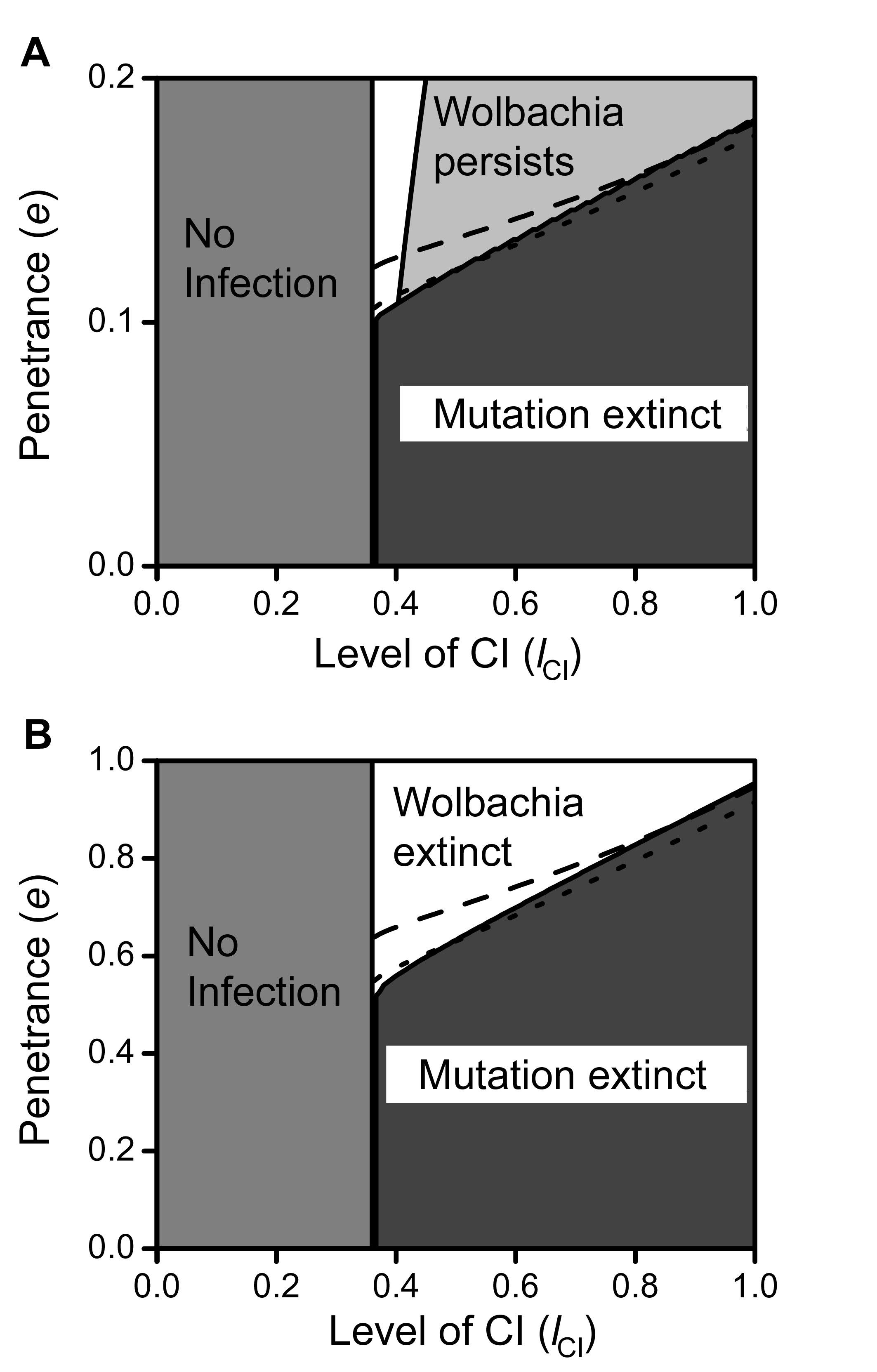


**Appendix Figure 1:** Shown are the parameter regions of a mutation's penetrance level *e* and the level of CI *l*_CI_ where a male-specific repressive mutation can spread in the population and how this affects the persistence of *Wolbachia*. Survival cost was set to *c* = 0.01 in 1a (scale of y-axis enlarged for clarity) and *c* = 0.05 in 1b. Analytical approximations for the threshold penetrance *e*_thr_ (as derived in section 'With mutants bearing survival costs' of this Appendix) are shown as dashed lines (one generation projected) or dotted lines (two generations projected). Other parameters were *t* = 0.9, *d* = 0, and *f* = 0.


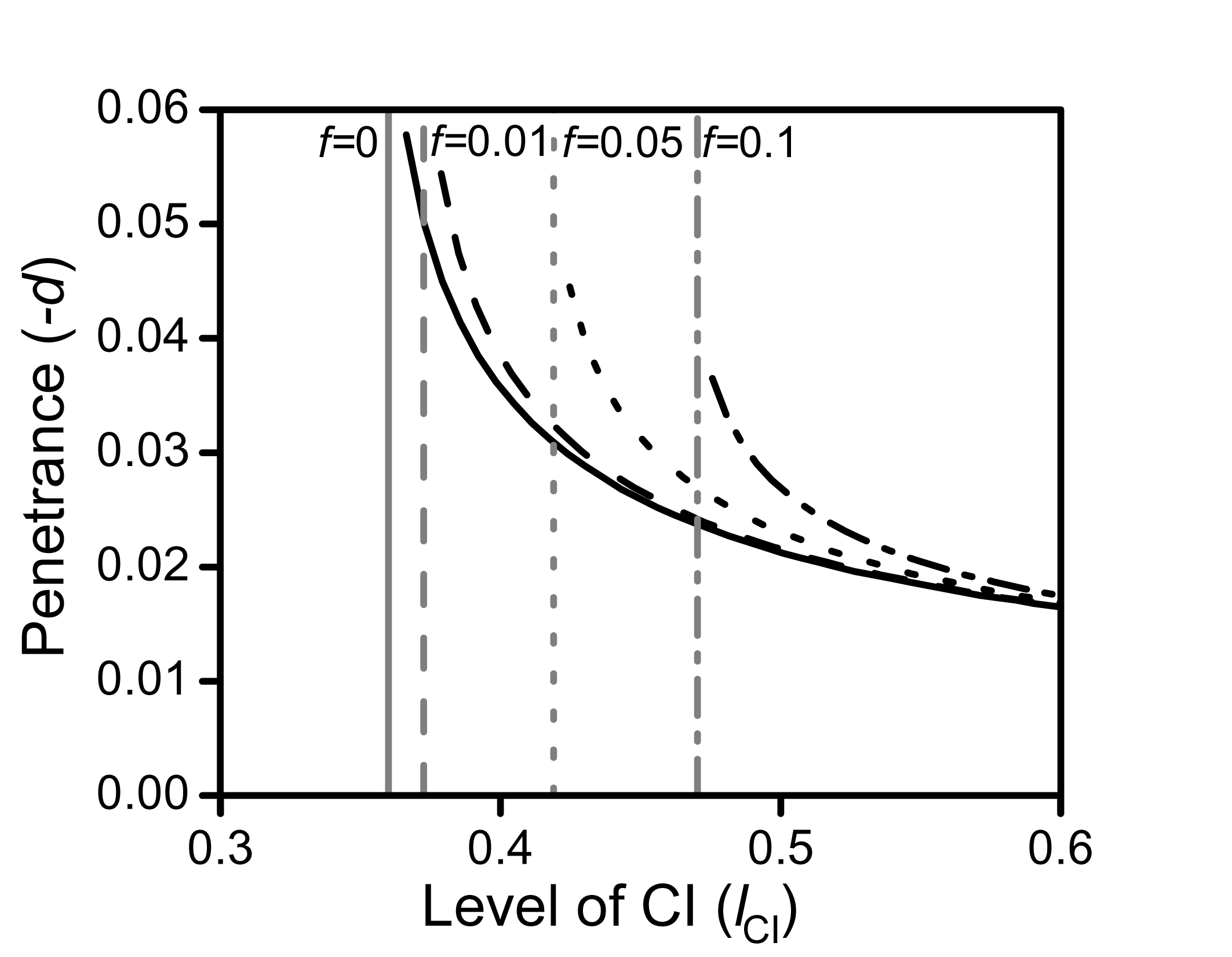


**Appendix Figure 2:** Shown are the parameter regions of penetrance levels -*d* and the level of CI *l*_CI_ where a female-specific enhancing mutation which increases transmission rates can spread in the population. The mutation invades above and goes extinct below the threshold lines indicated in black, while *Wolbachia*-infections cannot persist below the critical CI-levels that are indicated by grey lines. Fecundity costs *f* were varied between *f* = 0 (solid lines), *f* = 0.01 (dashed lines), *f* = 0.05 (dotted lines), and *f* = 0.1 (dash-dotted lines) as indicated. Other parameters were *t* = 0.9, *e* = 0, and *c* = 0.01.
